# Maintenance of Melanophore Morphology and Survival Is *Cathepsin* and *vps11* Dependent in Zebrafish

**DOI:** 10.1371/journal.pone.0065096

**Published:** 2013-05-27

**Authors:** Lauren F. Clancey, Alisha J. Beirl, Tor H. Linbo, Cynthia D. Cooper

**Affiliations:** 1 School of Molecular Biosciences, Washington State University Vancouver, Vancouver, Washington, United States of America; 2 Department of Biological Structure, University of Washington, Seattle, Washington, United States of America; Western University, Canada

## Abstract

Here, we characterize a *Danio rerio* zebrafish pigment cell mutant (*melanophore integrity* mutant), which displays a defect in maintenance of melanophore and iridophore number. Mapping and candidate gene analysis links the *melanophore integrity* mutant mutation to the *vacuolar protein sorting 11* (*vps11^w66^)* gene. Quantification of *vps11^w66^* chromatophores during larval stages suggests a decrease in number as compared to wildtype siblings. TUNEL analysis and treatment with the caspase inhibitor, zVAD-fmk, indicate that *vps11^w66^*chromatophore death is caspase independent. Western blot analysis of PARP-1 cleavage patterns in mutant lysates suggests that increases in pH dependent cathepsin activity is involved in the premature chromatophore death observed in *vps11^w66^* mutants. Consistently, treatment with ALLM and Bafilomycin A1 (cathepsin/calpain and vacuolar-type H+-ATPase inhibitors, respectively), restore normal melanophore morphology and number in *vps11^w66^* mutants. Last, LC3B western blot analysis indicates an increase in autophagosome marker, LC3B II in *vps11^w66^* mutants as compared to wildtype control, but not in ALLM or Bafilomycin A1 treated mutants. Taken together, these data suggest that *vps11* promotes normal melanophore morphology and survival by inhibiting cathepsin release and/or activity.

## Introduction

Zebrafish pigment cells or chromatophores develop from neural crest cells, a population of cells that arise along the dorsal aspect of the developing vertebrate neural tube early during development. Zebrafish stripes consist of black (melanophores), silver/iridescent (iridophores) and yellow (xanthophores) chromatophores. Together, these cells offer an intriguing system for studying cell migration, survival and differentiation. Additionally, melanophores have easy to track lysosomal related organelles, melanosomes. Melanosomes are critical for the synthesis of melanin, the characteristic dark pigment found in zebrafish melanophores and mammalian melanocytes. Thus, melanophores offer an additional model for examining genes and mechanisms involved in endocytosis, protein trafficking and protein/organelle turnover.

Protein/organelle turnover, or autophagy, is a cellular response to stress or toxic conditions leading to the collection and delivery of damaged proteins or organelles to lysosomes for degradation [1], [2]. Delivery occurs by collection of specifically selected proteins/organelles into double membrane structures called autophagosomes. Autophagosomes fuse with lysosomes for breakdown and degradation of contents. Autophagy depends on at least thirty three genes (Autophagy-related or ATG genes) in yeast, and many of these genes are conserved in higher eukaryotes (reviewed in [3]).

Along with ATG proteins, vacuole protein sorting (Vps) proteins are also critical for autophagy, as well as endolysosomal traffic, serving as tethers for vesicle fusion. Originally identified in yeast, two multisubunit complexes, HOPS (homotypic fusion and protein transport) and CORVET (class C core vacuole/endosome tethering complex), consist of a core subunit of Vps11, Vps18, Vps16 and Vps33, all of which are conserved in eukaryotes [4]. The class III phosphatidylinositol 3-kinase complex I is part of the core machinery necessary for autophagy to occur and includes several ATG proteins, along with Vps34 and Vps15 [5]. Recent evidence suggests a role for Vps18 in *Caenorhabditis elegans* (*C. elegans*) apoptotic corpse degradation [6], a process previously shown to be dependent on autophagy during embryonic development [7]. In melanocytes specifically, a genome wide screen followed by siRNA analysis uncovered several genes (including Wipi 1 and LC3-C) that function in both melanin synthesis and autophagosome formation [8]. Thus, characterization of *vps*/melanophore pigmentation mutants provides a powerful model for understanding the connection between Vps proteins, autophagy, cell death and melanogenesis. Here, we describe the effects of Vps11 protein loss on these activities in melanophores.

Mutations in *vps11* lead to several trafficking and pigmentation phenotypes in multiple organisms. *vps11* null mutations cause endosomal/vacuolar (homologous to mammalian lysosomes) delivery defects in yeast [9]. *vps11* function has also been analyzed in model organisms, *Oryzias latipes* medaka [10] and most recently, in *Danio rerio* zebrafish [11]. Although *vps11* mutations are lethal in both organisms, larvae live long enough to examine pigmentation defects. Loss of *vps11* leads to reductions in eye/body pigmentation and retinal abnormalities, illustrating the importance of this gene in normal pigment cell or retinal pigmented epithelium cell number and function. While these studies clearly indicate a role of *vps11* in pigmentation and melanosome maturation, a mechanism for pigment cell loss remains unclear. As cell loss/death can occur via multiple pathways, including caspase-dependent (apoptosis, pyroptosis) and caspase-independent pathways (calpain/cathepsin dependent apoptosis or oncosis/necrosis [12], [13]), our goal was to characterize the *vps11* dependent mechanisms for promoting pigment cell survival. Here, we show that *vps11*works to promote chromatophore survival in zebrafish in a cathepsin-dependent, caspase-independent manner.

## Methods

### Ethics Statement

These experiments, involving the use of model organism *Danio rerio* zebrafish, were approved by the Institutional Animal Care and Use Committee at Washington State University, ASAF# 03848-017.

### Fish Rearing and Crosses

Wildtype fish were of the AB (ZDBGENO-960809-7) strain. Adult zebrafish were maintained on a 14-hr/10-hr light/dark cycle at 28.5°C. Embryos were acquired from natural crosses, grown at 28.5°C in embryo media until analysis. Embryos were staged according to characterized morphological criteria [14]. The following alleles were used: *vps11^w66^*, *kit^w34^* [15] and WIK for mapping. *platinum* zebrafish were used for complementation and zVAD-fmk melanophore quantification analysis [11].

### Isolation, mapping and sequencing of melanophore integrity mutant

The *melanophore integrity/vps11^w66^* mutant was isolated as a spontaneous mutation arising in the zebrafish colony at the University of Washington. Heterozygous mutant *melanophore integrity* carriers in the wildtype (WT) AB background were outcrossed to the WT polymorphic WIK strain. Hybrid AB/WIK mutant carriers were intercrossed and mutant progeny were collected at 4 days post fertilization (dpf) when the phenotype became obvious. DNA from individual mutants and WT siblings was extracted by placing individuals in 50 µL of Lysis buffer (1.5 mM MgCl_2_, 10 mM Tris pH = 8, 50 mM KCl, 0.05% Tween-20, 0.05% Triton X-100) for 10 min. at 90°C, followed by a two hour incubation with 1 µL of 20 mg/mL proteinase K (Fisher Scientific, Pittsburgh, PA). The enzyme was deactivated by heating to 90°C for 10min and a 1∶20 dilution of DNA was used in further PCR reactions. Bulk segregant analysis was done on pooled DNA from 20 individual WT siblings and mutants. Microsatellite markers for each chromosome were amplified by PCR and mutant pools were evaluated for co-segregation. After establishing initial linkage to chromosome 10, fine mapping of recombination in mutant individuals (n = 926) was done using developed markers that amplified sequence length polymorphisms. The mutation was mapped to a 53 Kb region, flanked by primers:

- Primer 1 F: TGCACATGGTCTGAGGGTGGC
- Primer 1 R: GGTTGGGTGGGGTGAGGGCA
- Primer 2 F: CGTGCAGTCTGAAGCTCTCCGT
- Primer 2 R: TCACGTGTAGTAGTGCAACAGGAGT

For cDNA isolation and sequencing, RNA was isolated from pools of WT and mutant 6dpf whole larvae using TRIzol according to the manufacturer’s directions (Invitrogen, Carlsbad, CA). cDNA was synthesized using the First Strand cDNA Synthesis Kit (Invitrogen, Carlsbad, CA) with oligo-dT primer according to the manufacturer’s directions. WT sibling and mutant cDNAs were sequenced from overlapping PCR fragments averaging 500 basepairs by the Oregon Health and Science University Sequencing Core (Portland, OR). Primer sequences provided upon request.

For RT-PCR, RNA was extracted as previously described from 3dpf and 6dpf WT and mutant embryos. cDNA was made as previously described and amplified using oligo-dT primer. Primer sequences available upon request.

### Pharmacological treatments, chromatophore counts and statistical analysis

zVAD-fmk (Enzo Life Sciences, Plymouth Meeting, PA), a general caspase inhibitor, was added to embryo media at 24 or 55 (∼2.3hpf) hours post fertilization (hpf; Westerfield, The Zebrafish Book) with 1% Dimethyl Sulfoxide (DMSO) at a concentration of 300 µM. The control solution consisted of 1% DMSO in embryo media. For bafilomycin A1 (Axxora, San Diego, CA) experiments, 5dpf larvae were placed in embryo media containing 25 nM of the autophagy and vacuolar-type H+−ATPase inhibitor [16]. For cathepsin inhibition (ALLM is also a calpain inhibitor), fish were reared in 10 µM ALLM (Merck Millipore, Darmstadt Germany) in embryo media from 3dpf to 6dpf (for western blot analysis) or to 7dpf (for cell quantification). 10 µM ALLM solutions were made from a 10 mM ALLM stock suspended in 100% ethanol and control fish were reared in 0.01% ethanol in embryo media.

For melanophore quantification, larvae were incubated in 5 mg/mL epinephrine (diluted in embryo media) which aggregates melanosomes (promotes viewing of individual cells), anesthetized in MESAB and fixed in 4% paraformaldehyde in phosphate buffered saline, pH 7.2 (PFA/PBS) at room temperature for 1−2 hours or 14−18 hours at 4°C. Fish were positioned on Petri dishes or glass slides using 3% methyl cellulose to facilitate counting (images were not used for counting). Cells with distinct, continuous boundaries were counted as one cell (also referred to as melanophore fragments as fragmentation increases as *vps11^w66^* mutant larvae develop). Unless otherwise indicated, melanophore counts in all experiments included the dorsal (head, trunk and tail) lateral and ventral (posterior to the cloaca) stripes. For iridophore quantification, fish were anesthetized in MESAB and fixed in 4% PFA/PBS. To illuminate iridophores in live and fixed fish, brightfield and fiber optic light sources were used. Unless otherwise indicated, iridophore counts in all experiments included dorsal stripe iridophores. Quantification was done using Nikon SMZ1500 or Leica M80 stereomicroscopes. For all experiments, quantification data shown is representative of at least three experiments, 5−8 fish per timepoint and condition. Cell count data was analyzed using student t test, one way ANOVA or two way ANOVA with Bonferonni multiple comparison post test (Graph Pad Prism version 5.00 for Windows, Graph Pad Software, San Diego CA and Microsoft Excel 2007 for Windows). The Bonferonni post test includes analysis of all genotype/treatment or genotype/timepoint combinations as appropriate. Results of statistical analysis are indicated or discussed in Figure s (using asterisks), Figure captions and in the results section.

### In Situ Hybridization and Imaging

For in situ hybridization, all fish were treated with 1X 1-phenyl-2-thiourea (Sigma, St. Louis, MO) which inhibits tyrosinase and melanin synthesis. Iridophores are smaller/duller in *vps11^w66^* mutants and were used for identifying mutant larvae. Embryos/larvae were fixed in 4% paraformaldehyde in phosphate buffered saline, pH 7.2 and processed using standard protocols. Digoxigenin-labeled probes for *dopachrome tautomerase* [17] and *purine nucleoside phosphorylase 4a* [18] have been previously described. All imaging was done using a Nikon SMZ1500 stereomicroscope equipped with a Digital Sight DS-Ri1 Digital Camera or a Leica DMI400B Inverted Microscope equipped with a DFC420C Digital Camera. All images were processed for contrast, brightness and color using Adobe Photoshop CS3 Extended Version 10.0. For transmission electron microscopy, whole zebrafish were fixed in 2% glutaraldehyde in 0.1 M PBS followed by post fixation in 2% osmium tetroxide. Samples were dehydrated in an ethanol series followed by 100% acetone, infiltrated with Spurr’s resin and polymerized overnight at 70°C. Thin sections (90 nm) were placed on formvar coated nickel grids and stained with 4% uranyl acetate and Reynold’s Lead before viewing. Viewing was done using a Philips CM 200 at 200 KV (FEI Hillsboro, OR), equipped with a Orius, Gatan Digital camera (Warrandale, PA), located at the Franceschi Microscopy and Imaging Center, Washington State University, Pullman WA.

### Melanophore TUNEL Analysis

TUNEL analysis was done using In Situ Cell Death Detection Kits (Roche, Indianapolis, IN) using a previously described adapted protocol [15]. Briefly, fish were fixed in 4% paraformaldehyde in phosphate buffered saline (PFA/PBS), pH 7.2, for 1−2 hours at room temperature or 12-16 hours at 4°C. Larvae were dehydrated in a methanol/tris buffered saline series (MEOH/TBS (1 M Tris, pH 7.5, 1.5 M NaCl)) and rehydrated in the MEOH/TBS, containing 2% Triton X-100 and 5% Tween-20 (TBSTT). Larvae were digested with proteinase K, re-fixed in PFA/PBS, washed in TBSTT, and then incubated in TUNEL enzyme/label mix according to the manufacturer’s instructions. Following the incubation, larvae were blocked in 150 mM maleic acid, 100 mM NaCl (pH 7.5) plus 2% Western Blocking Reagent (Roche, Indianapolis, IN) and incubated in anti-fluor-POD antibody (Roche, Indianapolis, IN; 1∶1000 dilution). Following several washes in maleic acid/NaCl buffer, larvae were incubated in TSA plus fluorescein solution (Invitrogen, San Diego, CA) to amplify TUNEL signal through fluorescein quenching melanin. Larvae were visualized and imaged using a Nikon SMZ1500 stereomicroscope. *kit^w34^* zebrafish have a null mutation in *kit* tyrosine kinase receptor and only lose melanophores prematurely due to increased apoptosis [15], [19]. *kit^w34^* zebrafish were used as a positive control for zVAD-fmk and TUNEL assays.

### Western Blotting and Densitometry Analysis

Larvae (n = 25) were lysed in lysis buffer (10 mM Tris HCl pH 7.4, 2 mM EDTA, 150 mM NaCl, 2% Tween-20 and 2% Triton-X) supplemented with Halt protease and phosphatase inhibitor cocktail (Thermo Scientific, Rockford, IL). 2X SDS sample buffer (126 mM Tris HCl pH 6.8, 20% glycerol, 10% β-mercaptoethanol, 7% SDS, 0.0025% Bromophenol Blue) was added to the homogenate and the samples were first boiled for 5 min., then sheared using a 19-gauge needle (BD, Franklin Lakes, NJ). Supernatant proteins were separated on 10% (or 16% for LC3B analysis) Tris-glycine precast gels (Invitrogen, Carlsbad, CA) for 1.5 hrs at 125V in 1X Running buffer (24.7 mM Tris, 252 mM glycine, 3.47 mM SDS; XCell *SureLock* Mini Cell (Invitrogen, Carlsbad, CA)), then transferred to PVDF membrane (Thermo Scientific, Rockford, IL) at 25 V for 1 hour in 1X Transfer buffer (24.7 mM Tris, 252 mM glycine, 20% methanol). The membrane was washed in phosphate buffered saline with tween-20 (PBST; 137 mM sodium chloride, 2.7 mM potassium chloride, 8.1 mM sodium phosphate, 1.76 mM potassium phosphate, 0.1% Tween-20, pH 7.4) and blocked for 1 hour in 5% dry skim milk in PBST. The blots were incubated in rabbit anti-parp-1 1∶2000 (Calbiochem, Darmstadt, Germany), rabbit anti-pan cadherin 1∶3000 (Sigma St. Louis, MO), LC3B 1∶4000 (Novus Biologicals, Littleton, CO) or goat anti-vps11 1∶1500 (Abcam Inc., Cambridge, MA) overnight at 4°C. Blots were washed 3 times for 30 min. in PBST, incubated in 1∶10,000 goat anti-rabbit or rabbit anti-goat HRP-conjugated secondary antibody (Jackson Immunoresearch, West Grove, PA) for 1 hr and washed again (3 times, 30 min. each). Blots were incubated in Pierce ECL Western Blotting Substrate (Thermo Scientific, Rockford, IL) for 1 min., wrapped in plastic wrap, and exposed to Gene Mate blue autoradiography film (BioExpress, Kaysville, UT). Autorad film was developed using Kodak GBX developer and fixer (Sigma Aldrich, St. Louis, MO) following manufacturer’s instructions. Western blot band density quantification was done using ImageJ (National Institutes of Health). All groups were first standardized against their respective loading control and then against the wildtype control group to determine the relative densities. Analyses were done in triplicate and significance was tested by one way ANOVA.

## Results

### Chromatophore number is reduced in melanophore integrity mutants

**Figure 1:**
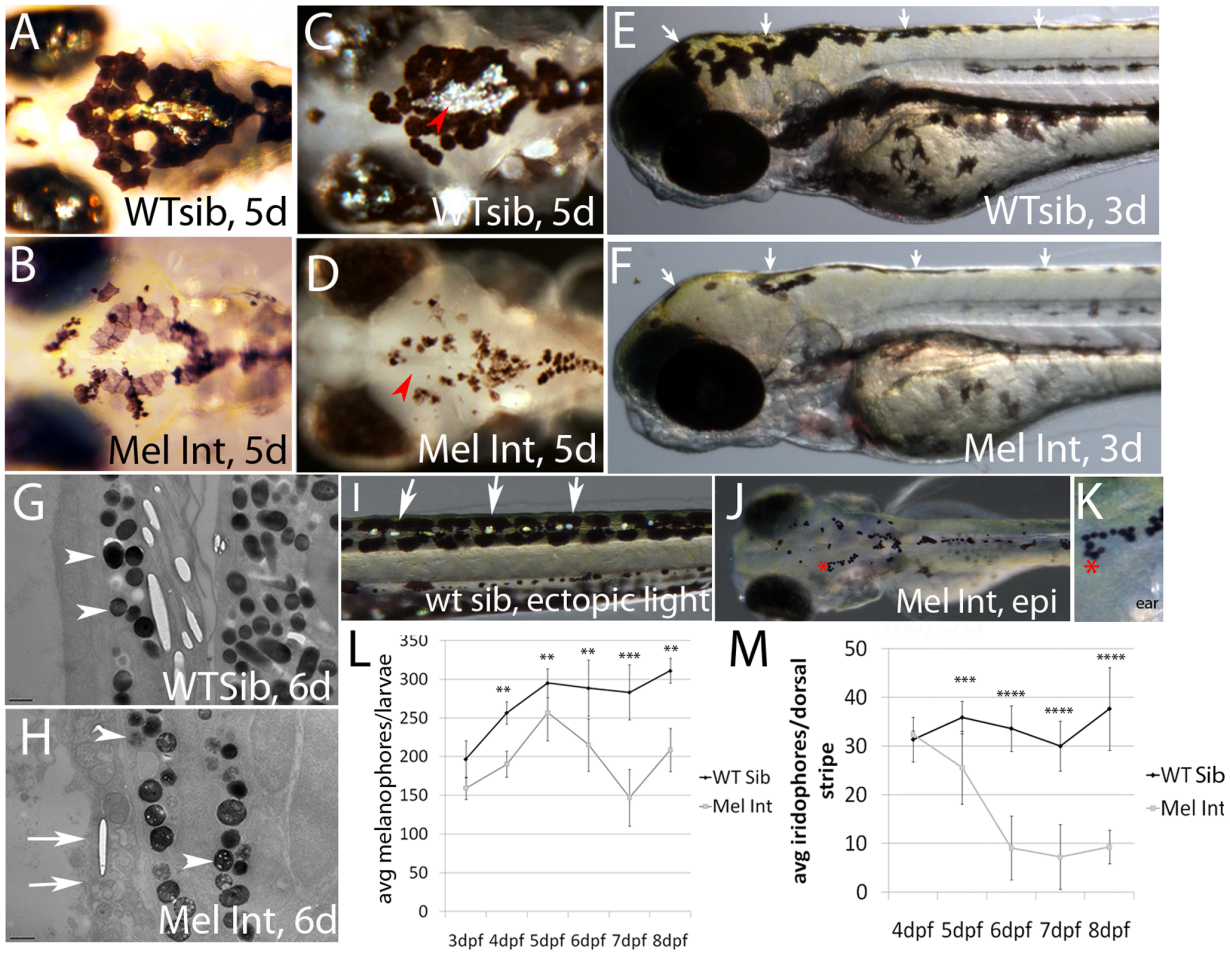
Melanophore *integrity* mutants show melanophore and iridophore defects. A, B) Dorsal, head images of live wildtype and mutant siblings, 5dpf. Wildtype melanophores maintain dark pigmentation and are now interacting with silver iridescent iridophores (present on eyes and in the center of caudal melanophore patch). Mutant melanophores are underpigmented and beginning to fragment. C, D) Dorsal image of caudal iridophore patch indicated by red arrow heads. Caudal patch and eye iridophores are the first to be affected in *integrity* mutants. E, F) Lateral image of 3dpf wildtype and *integrity* mutant larvae. We observe yellow coloration, indicative of xanthophores, in both wildtype and *integrity* mutant larvae (white arrows). G, H) Transmission electron microscopic images of 6dpf wildtype and *integrity* mutant larvae. Vertical sections were taken of dorsal stripe tissue and imaged at 3800X. *integrity* mutant melanosomes (compare arrowheads) are fewer in number and contain several presumed vacuoles as compared to wildtype. Epidermal plasma membranes are also fragmented (see white arrows) and internal organelle organization is disrupted. I-K) Representative images of fish used for quantification of iridophores (I) and melanophores (J, K). Trunk iridophores (in wildtype and mutant fish) are readily visible following epi-illumination using a fiber optic light source (see white arrows in I). Epinephrine promotes internalization of melanosomes, making individual melanophores easier to see and quantify (see red asterisks, which indicate the same region and is magnified in K). Distinct and continuous boundaries were used to designate a melanophore (e.g. the asterisk in K indicates four cells arranged in a U shape. L) Quantification of melanophores (dorsal and lateral stripes were included). Melanophores are significantly reduced as tested by two way ANOVA analysis with a Bonferonni multiple comparisons examining all genotypes and timepoints (p<0.001** or 0.0001***). M) Quantification of dorsal stripe iridophores in wildtype sibling and mutant larvae. Dorsal stripe iridophores are significantly reduced at all timepoints in mutant fish as tested by two way ANOVA analysis with a Bonferonni multiple comparisons examining all genotypes and timepoints (p<0.0001*** or 0.00001****). NOTE: 8−10 larvae per timepoint were counted; error bars are standard deviation.

We observe pigmentation defects in *melanophore integrity* mutants *(integrity)*, specific to iridophores and melanophores. At five days post fertilization (5dpf), *integrity* melanophores are fragmenting and showing variation in pigmentation levels (Figure 1A and 1B). Iridophores localized to the head regions are reduced (Figure 1C and 1D, red arrowheads) in *integrity* mutants. Examination of yellow coloration in 3dpf *integrity* mutants (Figure 1E and 1F, white arrows) suggests normal development of xanthophores (consistently, a yellow hue is still visible in 5dpf *integrity* mutants; Figure 1B). Using transmission electron microscopy, we examined dorsally localized melanophores in wildtype and *integrity* mutant fish at a magnification of 3800X, which allowed us to examine organelle organization in these cells. *Integrity* mutant melanophores show disruption in organelle morphology and structure as compared to wildtype (Figure 1G and 1H, white arrows). Additionally, the melanophore specific organelles, melanosomes, are irregular in morphology and contain presumed vacuoles (Figure 1G and 1H, arrowheads), suggesting defects in melanosome generation. To determine whether there was a reduction in *integrity* mutant melanophores as compared to wildtype, we quantified dorsal and lateral stripe localized melanophores in epinephrine treated, fixed fish (representative fish used for pigment cell quantification is shown in Figure 1I-1K; see methods for additional information). Quantification of these cells suggests a stall in the addition of melanophores in *integrity* mutants as compared to wildtype siblings, followed by a subsequent loss in cell number beginning at 5dpf (Figure 1L). Two way ANOVA analysis confirms a significant interaction between genotype and timepoint for melanophore number in *integrity* mutant fish as compared to wildtype siblings (p<0.004). Bonferonni multiple comparison analysis indicates a significant reduction in melanophore number in *integrity* mutant fish as compared to wildtype siblings (p<0.001** and 0.0001*** at each timepoint, 4-8dpf).

Trunk and tail iridophore numbers are also reduced in *integrity* mutants as compared to wildtype siblings. Quantification of iridophores (using a fiber optic light source, see Figure 1I) following ectopic illumination indicates a reduction in iridophore number beginning at 5dpf (Figure 1M). As seen with melanophores, two way ANOVA analysis indicates a significant interaction between genotype and timepoint for *integrity* mutant iridophore number as compared to wildtype (p<0.00002). Bonferonni multiple comparison analysis indicates a significant reduction in iridophore number as compared to wildtype (p<0.0001*** or 0.00001**** at each timepoint, 5−8dpf). Thus, *integrity* mutants have defects in melanophore and iridophore number.

### The melanophore integrity mutant harbors mutations in the vacuolar protein sorting 11 (*vps11*) gene

**Figure 2:**
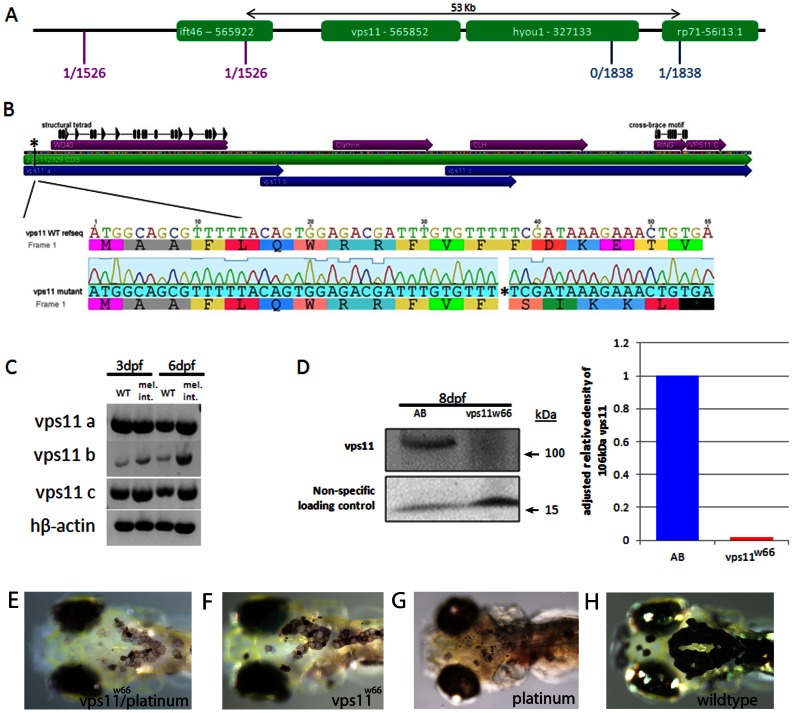
The melanophore integrity gene maps to a 53 kilobase region on chromosome 10. A) Flanking CA repeat markers and their respective number of recombinants per meioses (shown in red and blue) narrowed the region down to 53 kilobases, containing the *ift46*, *vps11* and *hyou1* genes. Being the most likely candidate (see text for details), *vps11* cDNA was generated and sequenced. B) Sequenced *vps11* cDNA indicated a single base pair deletion at base pair 37 which causes a frameshift and early stop at base pairs 53−55. Other differences in sequenced *vps11* cDNA include a single base pair substitution at base pairs 615 and 2183. C) RT-PCR on wildtype and *integrity* mutant mRNA indicates that *vps11* transcripts are present in *integrity* mutants. *vps11* cDNA was amplified in regions “a”, “b” and “c” as indicated in 2B (blue arrows). D) Immunoblot and densitometry analysis of Vps11 protein expression in wildtype (AB) and *vps11^w66^* mutant larvae. Vps11 protein is not expressed or is expressed at sub detection levels. E-H) Dorsal head images of live, 6dpf zebrafish larvae born from heterozygous *vps11* and *platinum* parents (E), heterozygous *vps11* parents (F and H) and heterozygous *platinum* parents (G). We observed the *integrity* phenotype in ∼25% of the clutch resulting from heterozygous *vps11* and *platinum* matings.

Using bulk segregant analysis, we linked the mutation to chromosome 10. Fine mapping analysis, narrowed the region to 53kilobases, containing the *ift46*, *vps11* and *hyou1* genes (Figure 2A). Previous studies examining *vps11* medaka and zebrafish (*platinum*) mutants indicated hypopigmentation phenotypes very similar to the *integrity* mutant [10], [11]. Based on these previous findings, we sequenced the *vps11* gene in mutant larvae. Sequence analysis indicates three distinct mutations: 1) a single base pair deletion at base pair 37 in exon 1 which leads to a predicted premature stop codon at residue 13 (Figure 2B). 2) a substitution at base pair 615 which leads to an amino acid change at residue 205 (E205D) and 3) a substitution at base pair 2183 which confers an amino acid change at residue 728 (K728R).

To check for exon splicing mutations, we designed primers to recognize overlapping regions of wildtype and *integrity* mutant*/vps11^w66^* for RT-PCR analysis. Human beta-actin was also analyzed as a loading control. *vps11* is expressed in *integrity* mutants as indicated by RT-PCR (Figure 2C). Because wildtype and mutant mRNA migrate at similar sizes on agarose gels, we predict that *vps11^w66^* mutants generate message of the correct size. However, western blot analysis for Vps11 protein in 8dpf larvae did not detect Vps11 protein (Figure 2D). Last, *platinum* failed to complement the *integrity* mutation in crosses with heterozygous *platinum* and *integrity* parents (we observed *integrity* phenotype in ∼25% of the clutch; Figure 2E−2H). Thus, it appears the *integrity* mutations in the *vps11* gene leads to generation of mRNA transcripts of predicted size. However, Vps11 protein is not generated (or generated in amounts below detection limit), suggesting the premature stop is responsible for the *integrity* phenotype.

### Examination of chromatophore specific gene expression indicates normal specification and migration in *vps11^w66^* mutants

**Figure 3:**
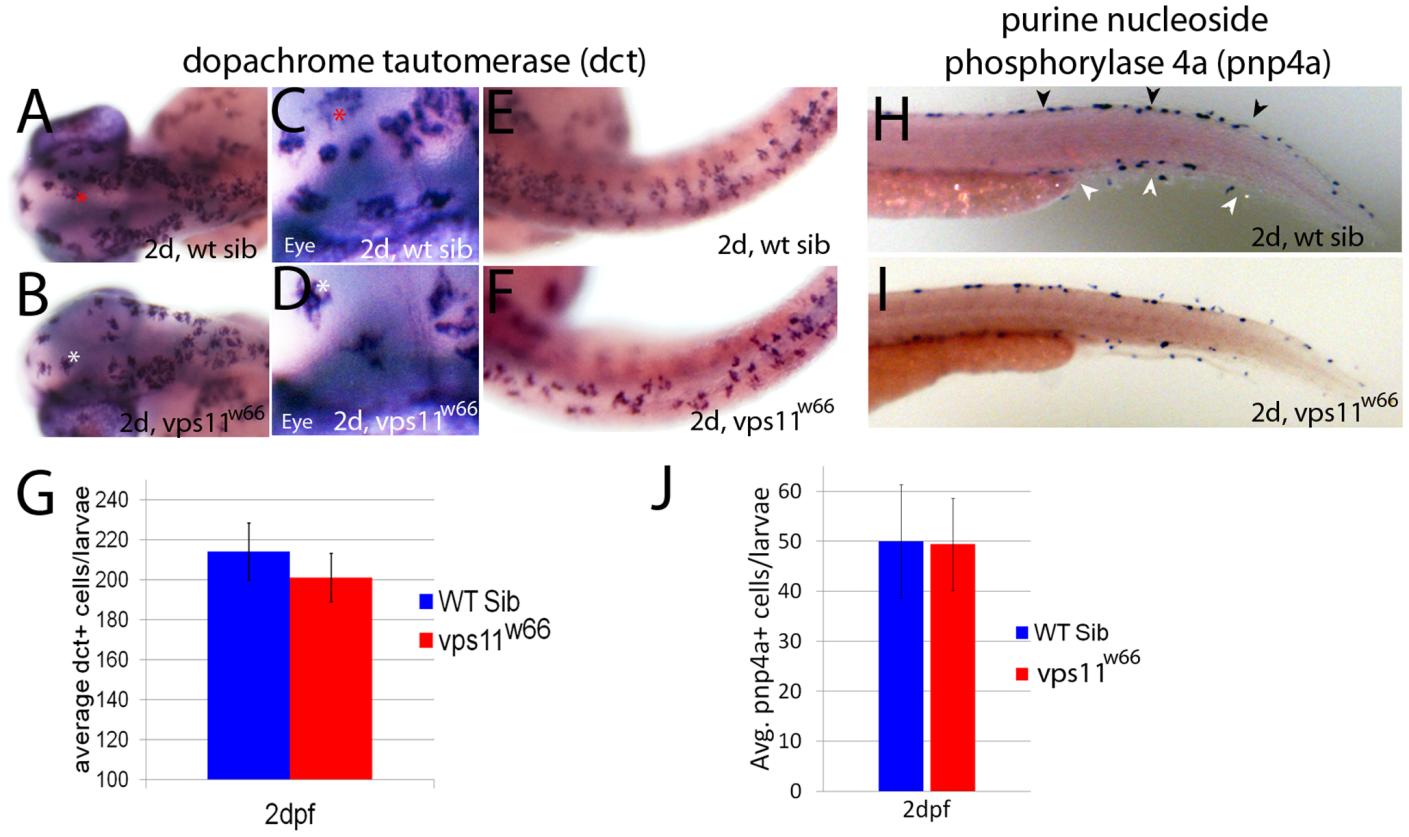
Chromatophore specific gene expression occurs at expected times and in wildtype levels in *vps11^w66^* mutants. A, B) Dorsal head images of 2dpf wildtype and *vps11^w66^* mutant larvae processed for melanophore specific *dopachrome tautomerase* (*dct*) expression. *dct* is detected in the correct place and time in *vps11^w66^* mutants. C, D) Close up examination of dorsal head regions shown in A and B. Asterisks indicate reference cells for orientation. Melanin synthesis was inhibited in all in situ hybridization experiments using 1X 1-phenyl-2-thiourea. E, F) Dorsal images of wildtype and *vps11^w66^* mutant trunk regions. In the trunk, *dct* is detected in expected levels but we observe uncharacteristic gaps between cells as compared to wildtype siblings. G) Quantification of *dct+* cells (dorsal, lateral and ventral stripes) suggests an insignificant reduction in cell number at 2dpf, consistent with pigmented melanophore counts in 1J (p = 0.14 via two way ANOVA with Bonferonni multiple comparisons analysis). Error bars are standard deviation. H, I) Lateral tail images of 2dpf wildtype sibling or *vps11^w66^* mutant larvae processed for iridophore specific *purine nucleoside phosphorylase 4a* (*pnp4a*) expression. Dorsal and ventral stripes are indicated in H by black and white arrowheads, respectively. J) Quantification of *pnp4a+* cells in 2dpf wildtype and *vps11^w66^* larvae suggest similar numbers of iridophore precursors at this stage (p = 0.85 via two way ANOVA with Bonferonni multiple comparisons).

*vps11^w66^* mutants, along with wildtype siblings (wt sib), were examined by in situ hybridization, using markers specific for melanophores and iridophores, *dopachrome tautomerase* (*dct*; [17]) and *purine nucleoside phosphorylase 4a* (*pnp4a*; [18]), respectively. 2dpf mutant and wildtype sibling larvae examined for *dct* expression, show normal head and trunk localization of *dct*+ cells (Figure 3A − 3F). Close up examination of dorsal head regions (Figure 3C and 3D) indicates similar cell morphology and intracellular *dct* distribution between wildtype and mutant larvae at this stage. Examination of trunk *dct+* cells suggests some differences in cell localization in mutant larvae, with mutant cell arrangement being slightly less organized (Figure 3E and 3F). Quantification of *dct+* melanophores suggests no significant difference between wildtype and mutant melanophores (Figure 3G; p = 0.14 via two way ANOVA analysis), suggesting that melanoblast specification from neural crest occurs normally in *vps11^w66^* mutants.

Wildtype and *vps11^w66^* larvae were also examined at 2dpf for *pnp4a* expression, a marker for iridophore differentiation ([18]; Figure 3H and 3I, dorsal and ventral stripes are indicated by black and white arrowheads, respectively). Iridophores are normally distributed in *vps11^w66^* mutants, populating both dorsal and ventral stripes at similar numbers between wildtype and *vps11^w66^* mutant larvae. Quantification of *pnp4a+* cells indicates no significant difference in cell number (p = 0.85 via two way ANOVA analysis at 2dpf) for wildtype and *vps11^w66^* mutants (Figure 3J). Taken together, this data suggests that melanoblasts and iridoblasts are specified in normal numbers from neural crest cells.

### Melanophore loss in *vps11^w66^* mutants is caspase independent

**Figure 4:**
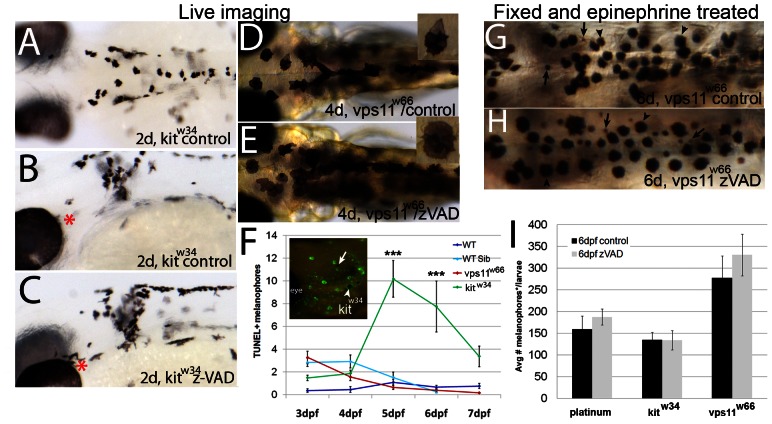
Melanophore loss in *vps11^w66^* mutants is due to a caspase-independent mechanism. A−C) Dorsal or lateral head images of 2dpf *kit^w34^* control (A, B) or zVAD-fmk (C) treated larvae, beginning at 1dpf. Red asterisks indicate regions of changes in melanophore morphology and number suggesting rescue following zVAD-fmk treatment. Quantification and student t test indicate a significant increase in melanophore number (*kit^w34^*control, 109±9; *kit^w34^*zVAD, 147±9 melanophores; p<<0.05 by student t test). D−E) Dorsal head and anterior trunk images (6X) of 4dpf (4d) control or zVAD-fmk treated *vps11^w66^* larvae. Inset: 11X magnification of a single melanophore from the same larvae, showing melanosome hyperaggregation and partial hypopigmentation. F) Quantification of TUNEL positive melanophores in wildtype (AB, dark blue), *vps11^w66^* wildtype sibling (cyan), *vps11^w66^* mutant (red) and *kit^w34^* mutant (green) zebrafish. We observe few to no TUNEL positive melanophores in *vps11^w66^* mutants at all timepoints, but a significant number of TUNEL positive melanophores in *kit^w34^* as expected. Two way ANOVA suggests a significant interaction between timepoint and genotype (p<0.0001). Multiple comparison analysis indicates significant differences in TUNEL+ cells between *kit^w34^* and all other genotypes at 5 and 6dpf (p<0.0001***). Inset: Lateral head image of TUNEL positive cells in *kit^w34^* mutant zebrafish (arrowhead, example of a TUNEL positive melanophore included in quantification; arrow, example of a TUNEL negative melanophore, not included in our quantification). Error bars are standard deviation. G−H) Dorsal trunk images of fixed 6dpf *vps11^w66^* larvae following control (G) or zVAD-fmk (H) treatment (2−6dpf; *vps11^w66^* phenotype first observed at 2dpf) and epinephrine treatment to promote melanosome aggregation to ease quantification. Similar to Figure 1, both melanophores (indicated by arrowheads) and presumed melanophore fragments (indicated by arrows) were counted. I) Quantification of dorsal, lateral and ventral stripe melanophores (*and fragments with continous boundaries) in *platinum*, *kit^w34^* or *vps11^w66^* control or zVAD-fmk treated larvae. Two way ANOVA analysis indicates no significant interaction for genotype and zVAD treatment (Note: zVAD-fmk treatment beginning at 1dpf is required for *kit^w34^* melanophore rescue).

Since melanophore specification and differentiation appear unaffected in *vps11^w66^*mutants, we examined increased apoptosis as an explanation for the gradual loss in cell number. As apoptosis is commonly caspase dependent, we tested the effects of zVAD-fmk treatment, a pan-caspase inhibitor, on *vps11^w66^* mutant zebrafish. *kit^w34^* mutants, containing a null mutation in the melanophore survival/anti-apoptosis gene *kit*, show premature melanophore death beginning at 2dpf [15], [19], [20]. As a positive control, we first tested the effects of zVAD-fmk treatment on *kit^w34^* larvae, beginning at 1dpf. Fish were examined by brightfield microscopy at 2dpf (Figure 4A−4C) or processed for melanophore quantification (epinephrine treated/fixed at 2dpf). While zVAD-fmk treatment has subtle effects on cell morphology in *kit^w34^* (Figure 4A−4C, red asterisks), it significantly increases positive control *kit^w34^* melanophore number as expected (*kit^w34^*control, 109±9; *kit^w34^*zVAD-fmk, 147±9 melanophores; p<<0.05 via student t test). Treatment of *kit^w34^* larvae with zVAD-fmk from 1-5dpf also significantly increased melanophore number as compared to control larvae (10 µM; *kit^w34^* control, 92±14; *kit^w34^* zVAD, 117±25 melanophores; p<0.05 via student t test).

To determine whether *vps11^w66^* melanophore death was caspase dependent, we treated larvae at 2dpf (when the *vps11^w66^* phenotype is first detected) and analysed via live imaging at 4dpf, a timepoint where cell fragmentation and hypopigmentation become readily apparent (Figure 4D). *vps11^w66^* melanophores showed similar morphology and melanin aggregation in control and zVAD treated larvae (4dpf; Figure 4D and 4E inset), suggesting a lack of rescue following zVAD-fmk treatment. To further examine the role of caspase dependent death in *vps11^w66^* melanophore loss, we examined these mutants using TUNEL assay. As TUNEL+ melanophores can be difficult to detect [15], [19], wildtype, wildtype sibling, *vps11^w66^* and *kit^w34^* larvae were examined at multiple timepoints, including time points before and after *vps11^w66^* melanophore loss begins (5dpf; Figure 1J). Consistent with initial zVAD-fmk results, TUNEL analysis out to 7dpf in wildtype, *vps11^w66^* wildtype sibling, *vps11^w66^* and *kit^w34^* larvae indicates an increase in TUNEL+ melanophores in *kit^w34^* mutants only (Figure 4F). We did not observe significant levels of TUNEL positive melanophores at any timepoint examined in *vps11^w66^* larvae. Two way ANOVA analysis indicates a significant interaction between genotype and timepoint for TUNEL+ melanophores (p<0.0001). Additionally, Bonferonni multiple comparison analysis, examining differences between means within each genotype, indicates a significant increase in *kit^w34^* TUNEL+ cells as compared to wildtype and *vps11^w66^* at 5 and 6dpf (p<0.0001^***^).

To confirm that *vps11^w66^* melanophore death is also caspase independent at later stages, we treated *vps11^w66^* larvae with zVAD-fmk from 2-6dpf and compared it to similarly treated *kit^w34^* and *platinum* mutants. Prior to fixation, all fish were treated with epinephrine to facilitate melanosome aggregation and quantification (representative images of quantified *vps11^w66^* larvae are shown in Figure 4G and 4H). Quantification of *vps11^w66^, kit^w34^* and *platinum* mutants indicates non-significant changes in melanophore number for all genotypes and treatment groups (Figure 4I; in our hands, *kit^w34^* rescue required zVAD-fmk treatment prior to 1dpf). Two way ANOVA analysis indicates no significant interaction between genotype and zVAD-fmk treatment. Taken together, this data is consistent with *vps11^w66^* larval melanophore loss being caspase independent.

### Bafilomycin A1 restores melanophore morphology and number in *vps11^w66^* mutants

**Figure 5:**
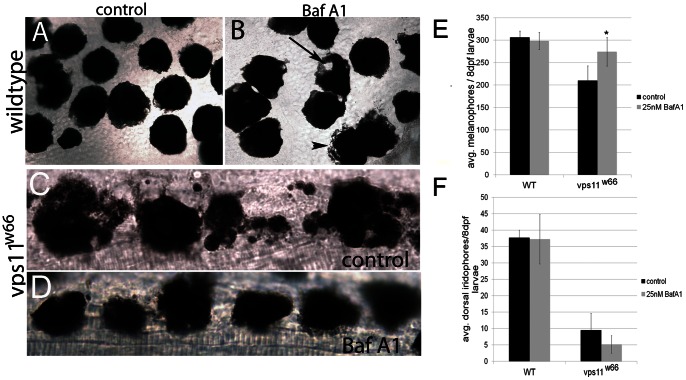
Treatment with bafilomycin A1 restores melanophore morphology and survival in *vps11^w66^* mutants. A) Dorsal head image (63X) of fixed, 8dpf wildtype larvae. Cells appear completely rounded, as expected following epinephrine induced melanosome aggregation. No vacuoles are observed. B) Dorsal head image of wildtype larvae treated with 25 nM bafilomycin A1 (Baf A1). Cell shape is less rounded, indicating reduced or incomplete melanosome aggregation (arrowhead). Large vacuoles are apparent (arrow). C, D) Dorsal anterior trunk images (63X) of *vps11^w66^* mutant melanophores treated with control (1% DMSO) or 25 nM Baf A1 in embryo media. Cell fragmentation and irregular cell morphology is partially rescued with Baf A1 treatment. E, F) Quantification of melanophores (dorsal and lateral stripes; E) or iridophores (F) in 8dpf wildtype and *vps11^w66^* mutant larvae treated with control (1% DMSO) or 25 nM Baf A1 embryo media. Only melanophores show significant increase in number following Baf A1 treatment. Two way ANOVA indicates a significant interaction between Baf A1 treatment and genotype. One way ANOVA and Bonferonni multiple comparisons analysis examining melanophore numbers within AB and *vps11^w66^* groups, indicates a significant increase in Baf A1 treated *vps11^w66^* larvae as compared to untreated controls (p<0.01*). Error bars are standard deviation.

Since chromatophore loss occurs gradually in *vps11^w66^* mutants, we became interested in examining the role of *vps11* in cell death delay mechanisms - including autophagy- using Bafilomycin A1 (Baf A1). Baf A1 specifically inhibits vacuolar-type H+−ATPase, leading to increases in lysosomal pH. Additionally, Baf A1 blocks lysosome/autophagosome fusion, a necessary final step in autophagy [21], [22]. For these experiments, wildtype (AB) and *vps11^w66^* mutant larvae were treated for three consecutive days in 25 nM Baf A1 beginning at 5dpf, the stage when melanophore loss begins (Figure 5A−5F). We chose this dose as it is higher than the 1 nM shown to be required for inhibition of autophagy in tissue culture cells [21]. Additionally, as cell death had already begun by 5dpf (as indicated by cell fragmentation and loss of plasma membrane integrity) and was past the previously characterized *kit* dependent 2dpf timepoint [20], we reasoned beginning treatment at 5dpf would also allow us to examine a role for *vps11* in the degradation/removal of melanocyte carcasses, a process previously shown to depend on Vps proteins and autophagy [6].

Prior to fixation at 8dpf, fish were treated with epinephrine to promote melanosome aggregation (images shown in Figure 5A−5D are of fixed individuals). Control wildtype melanophore melanosomes aggregate completely, appearing of equal size with few protrusions (Figure 5A). Conversely, control *vps11^w66^* mutant melanophores appear fragmented with small pockets of melanosomes surrounding larger, rounded fragments, further indicating disruption of normal cell morphology and plasma membrane integrity (compare Figure 5A to 5C). With Baf A1 treatment, a subset of wildtype melanophores appear partially aggregated (Figure 5B, arrowhead) whereas others have developed large vacuoles (Figure 5B, arrow), consistent with reduced lysosome fusion in response to Baf A1 treatment. Following Baf A1 treatment, *vps11^w66^* melanophore boundaries become more apparent, with fewer fragments (Compare Figure 5C and 5D), suggesting a reversal or delay of cell death as opposed to a delay in cell clearance. Quantification of control and Baf A1 treated wildtype and *vps11^w66^* mutant chromatophores at 8dpf indicates an increase in *vps11^w66^* treated larvae as compared to untreated (melanophores only; Figure 5E and 5F). Two way ANOVA analysis confirms a significant interaction between genotype and BafA1 treatment (p<0.0056). Additionally, Bonferonni multiple comparison analysis examining the effects of treatment within a specific genotype (AB and *vps11^w66^*) suggests a significant melanophore increase following Baf A1 treatment in *vps11^w66^* mutants only (p<0.01*). Thus, these data suggest that *vps11* protects melanophores from death by regulating autophagy, cytoplasmic pH and/or lysosome integrity.

### *vps11^w66^* mutants have greater levels of cathepsin activity

Our zVAD-fmk, TUNEL and Baf A1 data suggests that *vps11* promotes chromatophore survival in a caspase independent, pH dependent and/or autophagy dependent manner. To better understand the connection between autophagy, pH and caspase independent chromatophore death, we analyzed poly (ADP-ribose) polymerase -1 (PARP-1) cleavage in response to control (DMSO) and Baf A1 treatment. PARP-1 is an approximately 116 kDa nuclear enzyme that primarily functions to repair damaged DNA, consuming NAD in the process. In cells with increased and prolonged PARP-1 activity, proteases quickly cleave and inactivate the polymerase to keep ATP pools from becoming depleted [12], [23]. As PARP proteins are required for cell survival, they are cleaved by several enzymes when cell death is imminent. Protease cleavage of PARP-1 leaves characteristic molecular weight signature fragments, indicating caspase -dependent and -independent cell death [12], [23]. Caspase cleavage of PARP-1 during apoptosis has been well characterized in mammalian cell lines as well as more recently in zebrafish [12], [24], [25], where PARP-1 is cleaved into 89 and 24 kDa fragments.

**Figure 6:**
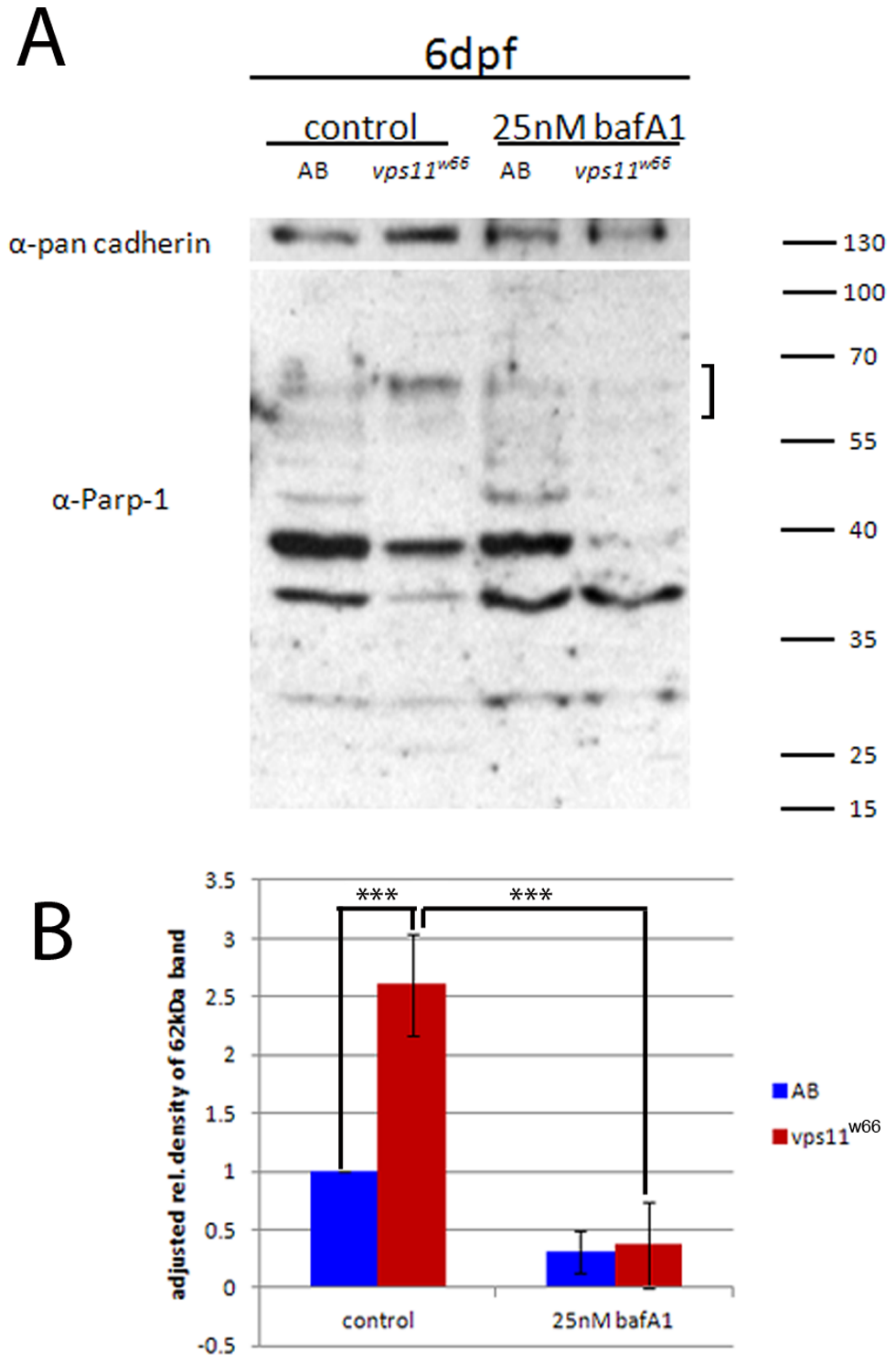
Increased levels of cathepsin protease activity are reduced by bafilomycin A1 treatments. A) Wildtype (AB) and *vps11^w66^* zebrafish lysates were analyzed by PARP-1 immunoblot and densitometry analysis. *vps11^w66^* lysates show higher levels of cathepsin cleavage products at 62 kDa in size as compared to wildtype fish. Treatment of larvae with Baf A1 reduces accumulation of the 62 kDa cleavage product. B) Graphical representation of density analysis of 62 kDa band from three immunoblots using Image J. Samples were analyzed via one way ANOVA with Bonferonni multiple comparison analysis (p<0.0001***). Error bars are standard deviation.

To better characterize the type of cell death occurring in *vps11^w66^* mutants, we analyzed PARP-1 cleavage. Specifically, we examined the accumulation of a 62 kDa band, which implicates cathepsin activity. This 62 kDa signature fragment, along with others at 44, 55 and 74 kDa, can be obtained from lysosomal proteases cathepsins B and D, and has been implicated in necrotic cell death in human cell lines [23]. *vps11^w66^* control fish show significantly higher levels of the 62 kDa PARP-1 cleavage product, which is reduced upon treatment with 25 nM Baf A1 (Figure 6A and Figure 6B; bracket). Anti-pan cadherin was used as a loading control. A 40 kDa cleavage product is thought to accumulate in response to increased calpain activity. However, we observed inconsistent variations in band intensities (within the 40 kDa range) making it difficult to analyze calpain activity using this assay.

**Figure 7:**
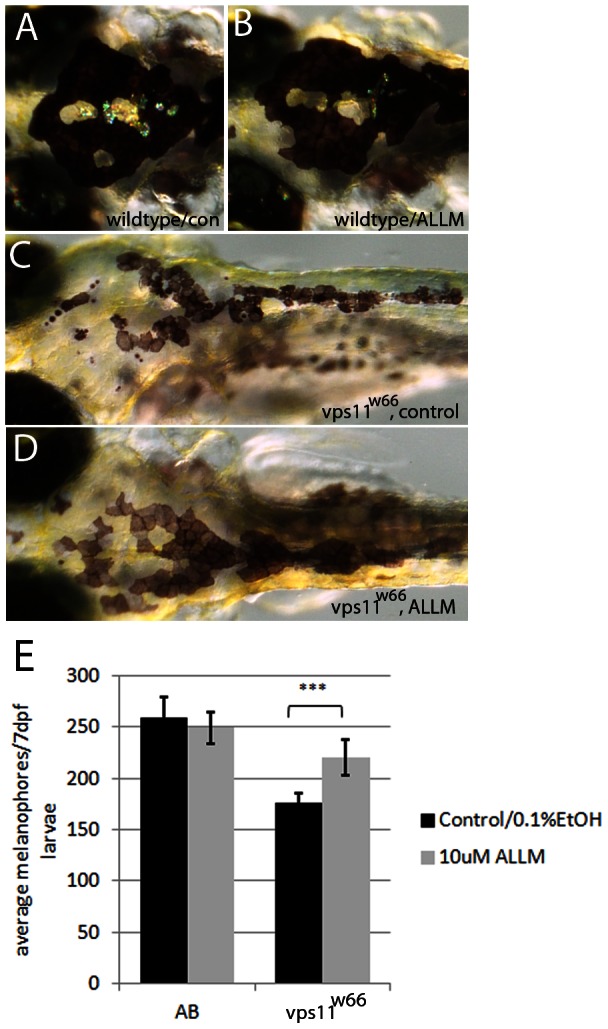
Calpain and Cathepsin inhibitor, ALLM, restores normal melanophore morphology and number. A−D) Dorsal images of (7dpf) wildtype or *vps11^w66^* larvae treated with 0.1% EtOH (A, C) or 10 µM ALLM (B, D) from 3−7dpf. Wildtype melanophore morphology remains unchanged, while *vps11^w66^* melanophores appear more uniform and less fragmented following ALLM treatment. E) Quantification of melanophores (dorsal and lateral stripes) in wildtype (AB) and *vps11^w66^* control or ALLM treated larvae. Note: two way ANOVA indicates a significant interaction between genotype and ALLM treatment (p<0.0001). Additionally, Bonferonni multiple comparison analysis examining effects of treatment within genotype groups suggests a significant increase in *vps11^w66^* melanophores following ALLM treatment (p<0.0001***). Error bars are standard deviation.

**Figure 8:**
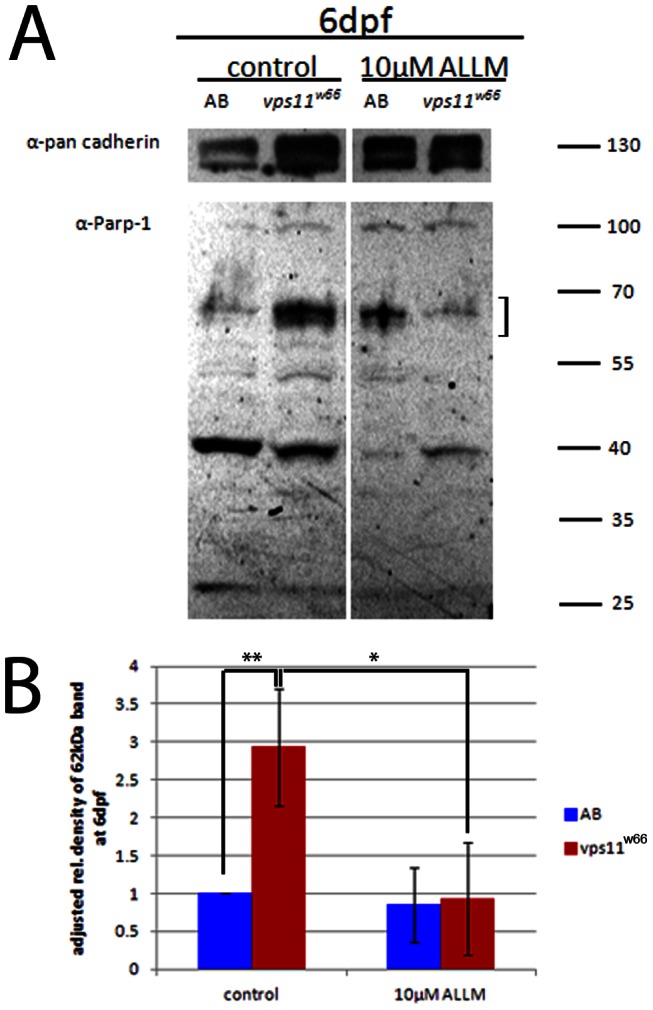
ALLM treatment reduces accumulation of 62 kDa PARP-1 fragment. A) Immunoblot analysis of PARP-1 cleavage fragments in wildtype (AB) and *vps11^w66^* larvae lysates. Treatment of larvae with 10 µM ALLM reduces the 62 kDa fragment (indicative of cathepsin activity; indicated by a bracket) below untreated levels. We also detect a 40 kDa cleavage product, which potentially represents calpain activity (see results text for additional details). B) Graphical representation of density analysis of 62 kDa band from three immunoblots using Image J. Samples were analyzed via one way ANOVA with Bonferonni multiple comparison analysis (p<0.05*, p<0.001**). Error bars are standard deviation.

To confirm a role for cathepsin in *vps11^w66^* dependent melanophore survival, we treated wildtype and *vps11^w66^* mutant larvae with ALLM, a cathepsin and calpain inhibitor. Treatment of *vps11^w66^* mutants (but not wildtype) with ALLM partially restored melanophore morphology and number in *vps11^w66^* mutants (Figure 7A-7E, significant interaction between genotype and ALLM treatment; p<0.0001). Bonferonni multiple comparison analysis examining effects of treatment within genotype groups indicates a significant increase in *vps11^w66^* melanophores following ALLM treatment (p<0.0001^***^). Western blot analysis confirmed these results, showing a reduction in cathepsin PARP-1 cleavage products (62 kDa, indicated by a bracket) following ALLM treatment of *vps11^w66^* larvae (Figure 8A and 8B; bracket). Other cathepsin dependent fragments, including the 44, 55 and 74 kDa bands, were not detected suggesting function of one specific cathepsin protein or fragment levels below our detection limits. Taken together, PARP-1 and ALLM analysis suggest an increase in cathepsin activity in *vps11* mutants, and that increases in cathepsin activity are detrimental to maintenance of melanophore morphology and survival.

### Autophagy is upregulated in *vps11^w66^* mutants

**Figure 9:**
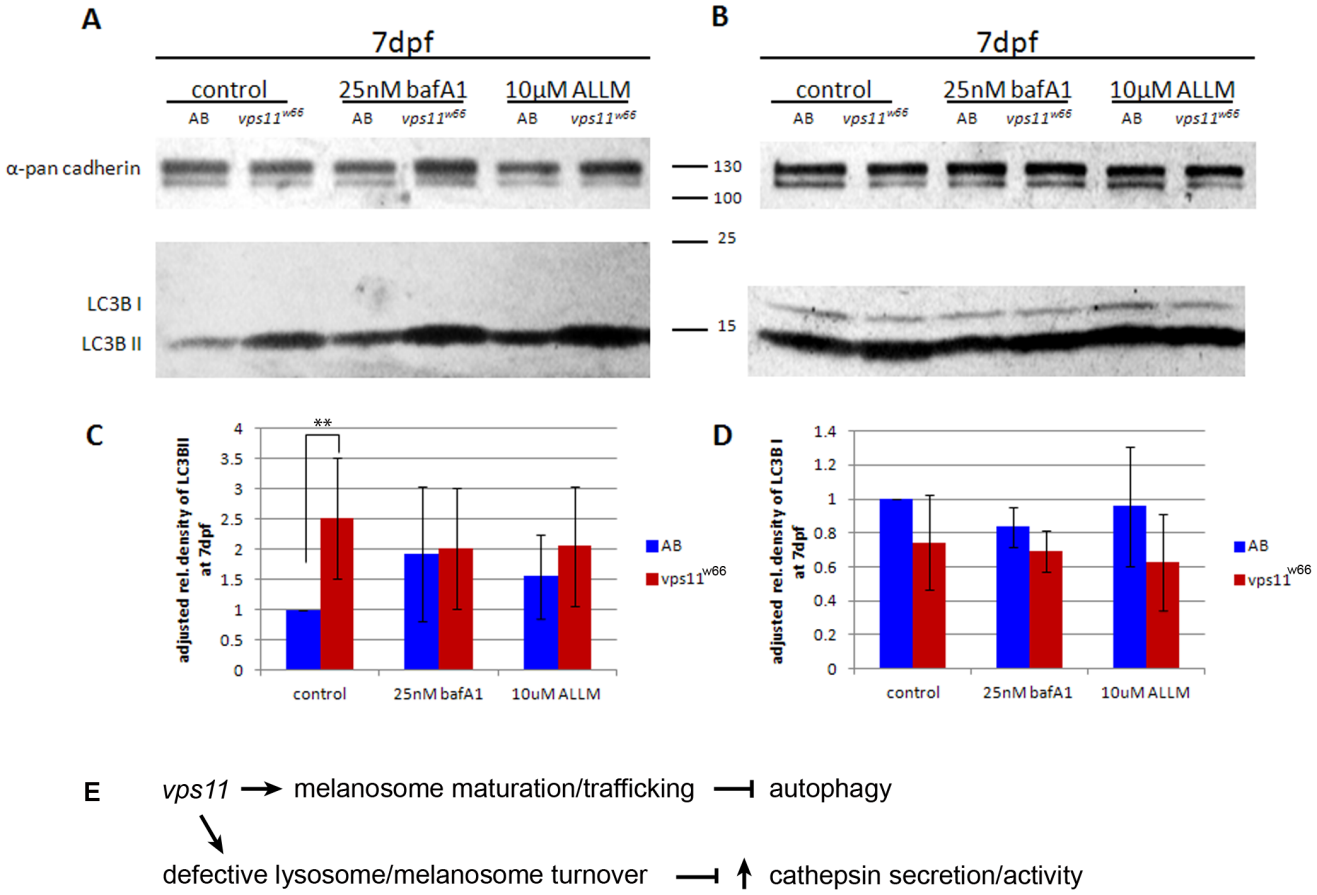
Baf A1 and ALLM treatment restore melanophore morphology and survival using an autophagy independent mechanism. A, B) Immunoblot analysis of LC3B II (A) and I (B) bands in control, Baf A1 and ALLM treated larvae lysates. A significant increase is detected in LC3B II in *vps11^w66^* larvae. We did not consistently observe an increase under Baf A1 and ALLM treatment conditions (see densitometry analysis). LC3B I levels were lower in mutants across treatments but not significantly different from control (1% DMSO or 0.01% ETOH) fish. Anti-pan cadherin was used as a loading control. C, D) Graphical representation of density analysis of LC3B II and LC3B I bands, respectively, from three immunoblots using Image J. Samples were analyzed via one way ANOVA with Bonferonni multiple comparison analysis (p<0.001**). Error bars are standard deviation. E) Model for *vps11* function in melanosome maturation, trafficking and integrity. We propose that *vps11* functions at dual locations to promote melanophore morphology and survival: 1) During melanogenesis to promote endosome/melanosome fusion and correct melanosome trafficking and maturation. 2) During autophagy to promote melanosome/lysosome/autophagosome fusion. When fusion does not occur at the latter step, organelle accumulation leads to the compromise of melanosome (and/or lysosome) integrity leading to increased cathepsin secretion and/or activity.

Because Baf A1 and ALLM treatment rescue melanophore number in *vps11^w66^* mutants, we examined the role of autophagy in Baf A1 and ALLM dependent rescue. To this end, we conducted immunoblot analysis of autophagosome marker, LC3B, in control, Baf A1 and ALLM treated *vps11^w66^* larval lysates (Figure 9A – 9D). LC3B can be detected as two bands, a cytosolic form (LC3B I) or an autophagosome bound form (LC3B II). Accumulation of LC3B II levels suggests an upregulation of autophagy and/or a block in autophagosome turnover [26]. In our hands, it is difficult to see both bands in the same immunoblot exposures. Therefore, two immunoblot exposures are shown, along with densitometry analysis for three independent experiments. Autophagosome membrane bound LC3B II levels were higher in *vps11^w66^* mutants across all treatments (Figure 9A−9D), consistent with the theory that mutations in c-class Vps proteins block autophagosome-lysosome fusion. Densitometry analysis confirms a significant increase in LC3B II levels only (p<0.001**; Figure 9A and 9C). However, there is no consistent/significant difference in LC3B I or II levels between *vps11^w66^* mutant treatment groups. This data indicates that autophagy is upregulated in *vps11^w66^* mutants and that pH, vacuolar-type H+−ATPase and/or cathepsin activity function downstream of or parallel to autophagy activation to promote melanophore survival.

## Discussion

Our results indicate a role for *vps11* in the maintenance of mature melanophore properties, including cell morphology, organelle integrity and survival. Iridophores also depend on *vps11*, showing a reduction in cell number at 5dpf. Similar to the zebrafish *platinum* and medaka *vps11* mutants, *vps11^w66^* larvae develop pigmentation at expected times, but later gradually lose melanophore and iridophore specific coloration (albeit more slowly than in *platinum* mutants). Additionally, *vps11^w66^* TEM experiments complements TEM analysis done in *platinum* retinal pigmented epithelium [11] by confirming melanosome maturation defects in melanophores. We also observe loss of plasma membrane integrity and accumulation of vacuoles in melanosomes. Close examination of individual melanophores using brightfield microscopy indicates cell fragmentation, indicative of cell death [13], [15], [27], [28]. TUNEL and zVAD-fmk analysis suggest cell loss occurs independently of caspase activity. Therefore, we examined other cell biological mechanisms to explain cell loss, including non-caspase dependent mechanisms.

Western blot and cell death analysis provide additional insight into the mechanisms underlying pigment cell loss in *vps11* mutants. PARP-1 immunoblot analysis confirms the accumulation of a PARP-1 62 kDa cleavage product, previously correlated with cathepsin activity [23]. Use of autophagy inhibitor/pH regulator, Baf A1, not only increases *vps11^w66^* melanophore number, but also reduces accumulation of the 62 kDa PARP-1 fragment, connecting *vps11* to regulation of intracellular pH and/or autophagy. Consistently, LC3 western blot analysis does indicate an upregulation of LC3B II in *vps11^w66^* larval lysates. Last, melanophore morphology and number are partially restored by ALLM (a cathepsin and calpain inhibitor) treatment. Similar to Baf A1, ALLM also reduced accumulation of the 62 kDa PARP-1 fragment. Taken together, these data suggest a model where *vps11* has dual function in melanophore maturation (Figure 9E). *vps11* promotes melanosome maturation and trafficking in melanophores, as previously proposed in zebrafish retinal pigmented epithelium [11]. Loss or downregulation of *vps11* activity results in accumulation of defective melanosomes, resulting in activation of autophagy. Additionally, we propose that *vps11* also promotes melanosome and lysosome integrity (possibly in an autophagy dependent manner). In the absence of *vps11*, accumulation of defective lysosomes and/or melanosomes leads to an increase in intracellular pH levels and cathepsin secretion and/or activity.

*vps11^w66^* mutants also have swim bladder and liver development defects (similar to *platinum*), suggesting that *vps11* serves chromatophore independent functions as well. In our PARP-1 analysis, we did not routinely observe other cathepsin dependent fragments, including 44, 55 and 74 kDa bands. Use of whole fish lysates for protein analysis opens the possibility of seeing variation in band intensities, especially when considering that other *vps11* dependent cells may utilize distinct mechanisms. The low intensity of the cathepsin bands (the 62 kD being the most abundant), along with the ALLM and Baf A1 rescue of melanophore number, is consistent with the theory that melanophores uniquely use a vps11/cathepsin dependent mechanism for controlling melanophore number in zebrafish. Although cathepsins have been previously implicated in melanocyte and melanophore differentiation/maturation [29], [30], our experiments provide insight into a role for *vps11* in modulating cathepsin activity. Further analysis is required to better define *vps11* and cathepsin function in non-melanophore *vps11* dependent cell types.

### Role for *vps11* in maintenance of melanophore number

Non-caspase dependent cell death mechanisms, such as necrosis, are characterized by loss of membrane integrity, clumping of chromatin, activation of cathepsin/calpain proteins, production of reactive oxygen species, ATP depletion and/or swelling of organelles depending on the system examined [13], [27], [28]. In zebrafish *vps11^w66^* melanophores, we observe lack of caspase dependent activity (TUNEL and zVAD-fmk analysis), an increase in cathepsin activity, swollen organelles, loss of organelle integrity and plasma membrane disruption, suggesting a necrotic phenotype in mutant melanophores. Additionally, Baf A1 treatment has been shown to inhibit necrotic cell death in *C. elegans*, suggesting a role for vacuolar H+−ATPase in the execution of necrosis [31] and a link to rescue of melanophore survival via Baf A1 treatment. Cathepsins are not just detected in lysosomes, but also in lysosome like organelles (possibly including melanosomes; [32]). Therefore, additional analysis is required to elucidate the source of increased cathepsin activity in *vps11^w66^* melanophores.

*vps11* function not only appears to affect iridophore and melanophore number, but also becomes less important at later larval timepoints. Subsequent chromatophore counts at 8dpf (Figure 1 and Figure 7) show an increase in melanophore and iridophore number in *vps11^w66^* mutants as compared to the previous timepoint. This occurrence may suggest a loss of dependence on *vps11* function as larvae progress towards juvenile stages, a phenomenon that has been shown for other melanophore specific genes (i.e. *kit, trpm7*; [19], [33]). As our *vps11^w66^*mutation is homozygous lethal, further investigation of temporal requirements for *vps11* during chromatophore development could be pursued using heat shock or temperature sensitive technologies to control the timing and extent of mutant allele expression.

### Role for *vps11* in inhibition of melanoma?

Autophagy has also been linked to melanoma cell survival. Oncogenic B-Raf (V600E) is associated with development of melanoma in humans and in 451Lu melanoma cells, its overexpression results in an upregulation of the autophagosome/autophagy marker, LC3B II. These cells also show a buildup of autophagosomes as detected by transmission electron microscopy [34]. It is unclear how melanoma cells activate autophagy and how it promotes melanomagenesis, however recent evidence suggests a leucine dependency [35]. Since our data suggests inactivating mutations in *vps11* lead to activation of autophagy, it is plausible that melanocytes containing these mutations are more susceptible to development of melanoma. Further examination of melanoma models in *vps11* loss of function backgrounds may shed light on mechanisms for activating autophagy during melanoma progression.
